# An App to Improve Eating Habits of Adolescents and Young Adults (Challenge to Go): Systematic Development of a Theory-Based and Target Group–Adapted Mobile App Intervention

**DOI:** 10.2196/11575

**Published:** 2019-08-12

**Authors:** Anna Rohde, Anja Duensing, Christine Dawczynski, Jasmin Godemann, Stefan Lorkowski, Christine Brombach

**Affiliations:** 1 Institute of Nutritional Sciences Faculty of Biological Sciences Friedrich Schiller University Jena Jena Germany; 2 Competence Cluster for Nutrition and Cardiovascular Health Halle-Jena-Leipzig Germany; 3 Department of Communication and Engagement in Agricultural, Nutritional and Environmental Sciences Justus Liebig University Giessen Giessen Germany; 4 Institute of Food and Beverage Innovation Zurich University of Applied Sciences Zurich Switzerland

**Keywords:** adolescents, young adults, mobile phone, mobile apps, mHealth, health behavior, healthy eating, motivation

## Abstract

**Background:**

Due to the widespread use of mobile phones, dietary mobile apps are promising tools for preventing diet-related noncommunicable diseases early in life. However, most of the currently available nutrition apps lack scientific evaluation and user acceptance.

**Objective:**

The objective of this study was the systematic design of a theory-driven and target group–adapted dietary mobile app concept to promote healthy eating habits with a focus on drinking habits as well as consumption of fruits and vegetables in adolescents and young adults, especially from disadvantaged backgrounds.

**Methods:**

The design process was guided by the behavior change wheel (BCW). The development process comprised 3 stages. In stage 1, the target behavior was specified, and facilitators and barriers were identified. Furthermore, important insights into target group interests, needs, and values in the field of nutrition and apps were revealed. To this end, 2 empirical studies were conducted with the target group. In stage 2, results of stage 1 were translated into behavior change techniques (BCTs) and, finally, into app functionalities and features. Consequently, in stage 3, the concept was evaluated and optimized through expert interviews.

**Results:**

Facilitators and barriers for achieving the target behavior were psychological capabilities (eg, self-efficacy), reflective motivation (eg, fitness), automatic motivation, social support, and physical opportunity (eg, time). Target group interests, needs, and values in the field of nutrition were translated into target group preferences for app usage, for example, low usage effort, visual feedback, or recipes. Education, training, incentives, persuasion, and enablement were identified as relevant intervention functions. Together with the target group preferences, these were translated via 14 BCTs, such as rewards, graded tasks, or self-monitoring into the app concept Challenge to go (C2go). The expert evaluation suggested changes of some app features for improving adherence, positive health effects, and technical feasibility. The C2go concept comprises 3 worlds: the (1) drinking, (2) vegetable, and (3) fruit worlds. In each world, the users are faced with challenges including feedback and a quiz. Tips were developed based on the health action process approach and to help users gain challenges and, thereby, achieve the target behavior. Challenges can be played alone or against someone in the community. Due to different activities, points can be collected, and levels can be achieved. Collected points open access to an Infothek (information section), where users can choose content that interests them. An avatar guides user through the app.

**Conclusions:**

C2go is aimed at adolescents and young adults and aims to improve their fruit and vegetable consumption as well as drinking habits. It is a theory-driven and target group–adapted dietary mobile intervention concept that uses gamification and was systematically developed using the BCW.

## Introduction

### Background

Globally, diet-related noncommunicable diseases (NCDs) are the leading cause of death and disease burden [1,2]. Numerous studies emphasize the association between a suboptimal diet and deaths due to NCDs such as stroke, heart disease, or type 2 diabetes [3-6]. Among dietary risk factors for NCDs are the low consumption of fruits and vegetables [7-9] and the high consumption of sugar-sweetened beverages [10-14].

German survey data highlights the high prevalence of overweight and obesity. Almost 60% of the population is overweight or obese (women 51% and men 66%) [15]. Among the younger population, about 16% of girls and 18.5% of boys in the age between 14 and 17 years are overweight or obese [16], likely due to a more sedentary lifestyle characterized by decreased physical activity and unbalanced dietary behavior [15]. Only 7% of the girls and boys in Germany in the age between 14 and 17 years follow the dietary recommendations for the consumption of 2 portions of fruits and 3 portions of vegetables each day. On average, 0.9 portion each of vegetables and fruits per day are consumed [17]. Nearly, 23% of boys and 17% of girls drink sugar-sweetened beverages daily [18], whereas recommended beverages are water and unsweetened teas [19]. Data from the United States also reveal similar results. American guidelines recommend 2.5 cups of vegetables and 2 cups of fruits per day (for a calorie level of 2000 kcal) as well as drinking water and reducing consumption of sugar-sweetened beverages [20]. However, less than 50% of children and adolescents meet dietary recommendations for any food group [21]. The level of education positively influences food consumption and, thus, the quality of the diet [22,23]. Higher school education and higher income result in a lower body mass index [15,16].

To summarize, nutrition surveys highlight that adolescents and young adults have unbalanced diets [17,24,25], especially adolescents and young adults in disadvantaged circumstances [17]. As adolescence is characterized by cutting ties with the parents’ household and the development of one’s own lifestyle, this could be a reasonable stage of life for behavior change interventions [26], in particular for focusing on adolescents and young adults with lower education levels.

### Digitalization and Mobile Health

State-of-the-art mobile phones provide new possibilities for dietary interventions. Mobile health (mHealth) is an emerging field and describes various health services offered on portable devices. These include health apps in various areas, such as nutrition, fitness, wellness, diagnostics, and therapy [27], but systematic studies in the area of mHealth are scarce [28-30]. Studies also highlight missing user acceptance of nutrition apps, for which the relatively high usage effort might be a reason [31,32]. Rohde et al concluded that app usage in the long term is influenced by user- and app-related acceptance factors [32]. The former highlights the importance of knowing the target group for designing accepted mHealth interventions; the latter emphasizes the importance of considering different app characteristics, for example, implementing instructions or motivators for engagement and adherence in app-based interventions [32].

In the context of long-term adherence and acceptance of mHealth interventions, *gamification* is an emerging field. Gamification means that playful elements such as points or leaderboards are used in a context that is normally not played (for example using a quiz, where one can earn points, instead of just giving information) [33,34]. Gamification can be a motivational component of digital behavior change interventions by playfully making uninteresting topics interesting and, thereby, engage users in the long term [34].

### Theory Guidance for Intervention Development

The behavior change wheel (BCW) is a framework for developing health interventions [35]. It proposes a systematic design process of behavior change interventions that helps to translate theory into practice [30].

The health action process approach (HAPA) is a health behavior model and, as a stage model, an interesting template for the theory-based development of dietary health messages that can be adapted to persons at different stages of the behavior change process [36]. It was successfully applied in several nutrition behavior change interventions [37-39].

### Objective

The aim of this study was to describe the iterative concept development and the final concept of a theory-based and target group–adapted mobile app for motivating adolescents and young adults (aged 14-25 years), especially from disadvantaged backgrounds, to improve their dietary habits with respect to the consumption of fruits and vegetables, as well as drinking behavior.

## Methods

### Overview

**Figure 1:**
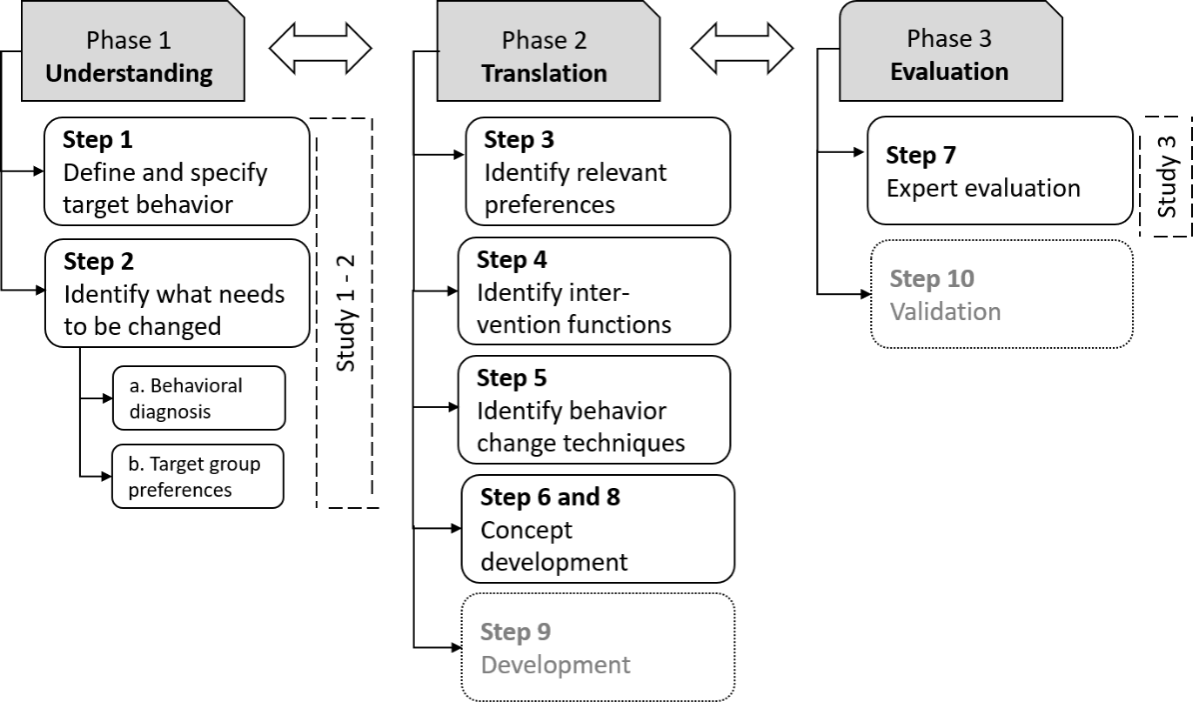
Systematic design process of the dietary mobile app for adolescents and young adults. Steps 1-7 (in black font) are discussed in the text; steps 9 and 10 (in grey font) are being or will be carried out.

The app design process was guided by the BCW [40]. After defining the problem and the 3 target behaviors, the app design process followed 3 stages (Figure 1): Phase 1, specifying the target behavior and identifying what needs to change to achieve it; Phase 2, translating results into app functionalities and features; and Phase 3, expert evaluation of the concept. In total, 3 empirical studies were conducted to derive relevant app features and content as well as to optimize the concept.

### Phase I: Understanding Behavior and Target Group Preferences

#### Step 1: Define and Specify Target Behavior

In total, 3 target behaviors were chosen by the author (AR) by identifying possible target behaviors through literature search and opinions of nutrition experts from the Friedrich Schiller University Jena. A list of potential target behaviors was shortened using the following criteria: Likely impact of the target behavior on outcome, ease of reaching target behavior, possible positive or negative spillover effects, and ease of measurement [40]. Each criterion was rated as unacceptable, unpromising but worth considering, promising, or very promising. Rating and selecting the target behavior were supported by empirical research (Step 2b). Upon selection of the most promising target behaviors, they were specified in detail and context: who, when, where, how often, and with whom will the target group perform the target behavior?

#### Step 2: Identify What Needs to Change to Reach Target Behavior

##### Behavioral Diagnosis

The capability-opportunity-motivation-behavior (COM-B) model was used to identify what needs to change for adolescents and young adults to achieve the target behavior. Evidence from the literature and empirical research informed this procedure by exploring target group’s capabilities, motivation, and opportunity to achieve the target behaviors (eg, which physical capabilities are needed to eat 2 portions of fruit per day?) and the need for change. Next, each capability, motivation, and opportunity was evaluated as feasible or not for implementing in a dietary mobile app.

##### Target Groups Preferences (Sampling From Empirical Studies 1 and 2)

Besides informing behavioral diagnosis, study results were also analyzed to explore the target group’s preferences for app characteristics, behavior change techniques (BCTs), features, and content. The ethics committee of the University noted no ethical concerns (processing number 4850-06/16).

###### Study 1: Nutrition and Apps From the Target Group’s Perspective

The objective of this study, conducted in 2016, was to get insights into nutrition habits, values, and needs, and to develop ideas as to how nutritional behavior and health among adolescents and young adults could be improved through mobile apps. Study participants (n=11) tested the German dietary mobile app *Was ich esse* [41] for 1 week before face-to-face, semistructured interviews. The participants were between 14 and 21 years old (average age: 18 years, SD 2.4) and mostly women (n=8). A total of 5 participants went to secondary school at the time of the study. Others were university students (n=3), trainees (n=1), volunteers (n=1), or were looking for an apprenticeship (n=1). The audio-recorded material was transcribed and analyzed by means of content analysis [42]. Data segments were coded into the following topics: (1) mobile phone and app usage, (2) test app experiences, (3) nutritional habits, (4) nutrition values, (5) nutritional improvement wishes and strategies, and (6) understanding of health. Additional information on recruitment, compensation, the interview guide, and the test app are provided in Multimedia Appendix 1.

###### Study 2: Nutrition and Mobile Phone Apps—Interests, Needs, and Values Among Adolescents and Young Adults

This study aimed for a better understanding of available mobile phone resources, app use, and needs as well as interests and values in the field of nutrition of adolescents and young adults. To this end, a questionnaire was developed and administered as a paper-pencil version. It included the following topics: mobile operating system, mobile phone rate, favorite apps, experiences with dietary mobile apps, importance of different app characteristics (eg, importance of customizability), and nutritional interests (eg, sports nutrition, health, and food waste) and values (eg, freshness of food and self-cooked meals). Data were analyzed descriptively. The inclusion criterion for participation was an age between 14 and 25 years. Teachers and social workers in Jena were contacted via mail to secure them as gatekeepers. In total, 210 participants from 5 different organizations took part (females n=99, males n=108, no information provided n=3). The average age was 18 years (n=208, SD 2.4; no information provided n=2). The youngest and oldest person was 15 and 25 years old, respectively. Participants went to vocational school (Berufs(fach)schule) (n=164). Others stated that they had participated in vocational preparation classes (n=27) or went to high school (Gymnasium) (n=11). One person each went to secondary school (Hauptschule) and regular school (Regelschule); 4 persons stated *other* (no information provided n=2). Additional information on recruitment, compensation, and the scales in the questionnaire are presented in Multimedia Appendix 1.

### Phase II: Translation of Research Results Into App Content and Features

#### Step 3: Identify Relevant Target Group Preferences

**Figure 2:**
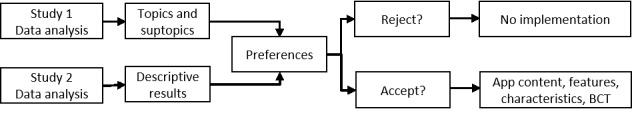
Process of identification of relevant target group preferences. BCT: behavior change technique.

Results from studies 1 and 2 were compared and merged in target group preferences, and an acceptance-rejection process followed (Figure 2). Decisions of the author (AR) for rejection or acceptance of target group preferences were based on APEASE criteria [40]: Is the respective preference *a* ffordable, *p* racticable, *e* ffective, and *a* cceptable, are *s* ide-effects expected or offense against *e* quity? If all criteria are met or at least rated probably, the preference was accepted and further implemented as app content, app features, BCTs, or considered as important app characteristics (Steps 5 and 6).

#### Step 4: Identify Intervention Functions

This step aimed to move from understanding the behavior to selecting intervention functions. This was supported by a matrix of links between COM-B and intervention functions [40]. Appropriate intervention functions were selected by using the APEASE criteria.

#### Step 5: Identify Behavior Change Techniques

To choose which BCTs can deliver the intervention functions, a linking was used [40]. The list of candidate BCTs (n=118) was narrowed by APEASE criteria. The rating was supported by the evidence of effectiveness for promoting healthy food choices, and on the basis of target group preferences.

#### Step 6: Concept Development (Prototype I)

Together with target group preferences, the BCTs were translated into app features, content, and characteristics. Content development of feedback was guided by HAPA for preintender and intenders and gamification aspects were implemented to enhance user engagement with the app [30,34].

### Phase III: Evaluation

#### Step 7: Expert Evaluation

The concept was evaluated and optimized using 3 evaluation criteria: (1) acceptance among target group, (2) positive health effects due to app use, and (3) technical feasibility. To this end, professionals with knowledge of the target group, app development or nutrition behavior were recruited. Recruitment took place via emails and later personally by telephone. Ultimately, 8 face-to-face interviews were conducted with experts of the following professions: Marketing, 2 social workers/teachers, dietician, app development, media psychology, psychotherapist, and a person of the target group itself. The interviews started with the presentation of the concept using mock ups. The semistructured interviews were audio recorded and transcribed verbatim. Data were evaluated with structured qualitative content analyses [42].1 The following 5 topics were discussed: (1) important features and needs of a dietary mobile app among the target group, (2) advantages of the concept, (3) disadvantages of the concept, (4) suggestions to improve the concept, and (5) technical feasibility.

In the next step, the recommendations for improving the concept were rated using APEASE criteria and were either accepted and implemented, or rejected and not implemented.

### Step 8: Final Intervention (Prototype II)

On the basis of the evaluation, the concept was adapted by defining the final features and functionalities of the app.

## Results

### Phase I: Understanding Behavior and Target Group Preferences

#### Step 1: Defined and Specified Target Behavior

A range of target behaviors was listed, such as consumption of more vegetables, fruits, water, tea, fibers, or eating less saturated fatty acids. Following this, an important point in rating the ease of achieving the behavior was to avoid the feeling of waiver in nutritional terms, so that a target behavior does not forbid, but permits and increases, consumption of food [43].

Finally, 3 target behaviors, in line with German intake recommendations [19] were chosen: The consumption of (1) 2 portions of fruits per day, (2) 3 portions of vegetables per day, and (3) drinking 1.5 L or more of unsweetened beverages per day to decrease the consumption of sugar-sweetened beverages (only nonalcoholic beverages are considered). To achieve the target behaviors, adolescents and young adults have to eat and drink fruits, vegetables, and sugar-free drinks at mealtimes (or in between), and often enough to achieve the respective target behavior. They can do it anywhere and either by themselves or with others.

#### Step 2: Identified What Needs to Change to Reach Target Behavior

##### Behavioral Diagnosis

The results of the behavioral diagnosis revealed facilitators and barriers to the target behavior in the following COM-B components: psychological capabilities (eg, nutrition education, self-efficacy, and risk perception), reflective motivation (eg, weight loss, satiety, fitness, and illness prevention), automatic motivation, social support, and physical opportunity (eg, time and financial resources). An overview of the results with quotes from study participants and references is displayed in Multimedia Appendix 2.

##### Target Group Preferences: Empirical Study Results

###### Study 1

An excerpt of the results in 4 of the 6 main topics is presented in Table 1. In addition, Multimedia Appendix 3 presents a complete overview of the results.

###### Study 2

The operating system most used was Android (Google) and most of the participants used a mobile flat rate. Among the participants’ favorite apps were communication and social media apps (WhatsApp, Facebook, and Instagram), video apps such as YouTube, and gaming apps such as Clash of Clans and Clash Royal. In all, 26% of the participants had experiences with apps in the area of nutrition, above all recipe apps. The most interesting subjects in the area of nutrition were health, cooking, and sports nutrition. Good taste, satiety, and freshness of food were the most important nutritional values. The most important app characteristics were free of charge, contact to friends/family, and fast use. Multimedia Appendix 3 gives a full insight into the results.

### Phase II: Translation of Research Results Into App Contents and Features

#### Step 3: Identified Relevant Target Group Preferences

An excerpt of results of the process of the identification and selection of relevant target group preferences for app characteristics and features is presented in Table 2. For the derivation of the preferences, all subtopics of the topics (1) to (5) of study 1 were included as well as the most frequent answers of study 2. Results that were rated mostly *not important* and *partly true* with tendency to *not important* were not considered to derive preferences. For example, *Nutrition habits of other cultures* and *Nutrition and skin* were both mostly rated as *partly interesting* (n=91; n=95). The first was not considered to derive a preference because it shows a tendency toward *not interesting* (n=72). However, the latter showed a tendency toward *interesting* (n=65) and was therefore considered to derive a preference.

#### Step 4: Identified Intervention Functions

Candidate intervention functions were education, persuasion, incentivization, coercion, training, restriction, environmental restructuring, modeling, and enablement. The rating of theses for both fruits and vegetables, and drinking behavior led to the selection of education, persuasion, incentivization, training, and enablement.

#### Step 5: Identified Behavior Change Techniques

According to the APEASE criteria, 14 BCTs were derived to bring about behavior change (Table 3).

**Table 1:** Excerpt of results from study 1.

Main topic, subtopics		Quotes (translated)
**Mobile phone and app usage**		
	Mobile phone is used for entertainment and when bored (eg, games and videos)	Jana: “I use my mobile phone when I'm bored or when I have to wait for the bus or something, then I play games.”
**Nutritional values**		
	Cooking stands for independency	Caro: “Yeah, for later, if maybe I have a family and I cannot cook, that would be a bit ... And cooking is also important to me, so I do not always depend on someone.”
	Spending on food should be kept low	Leon: “We like to eat exotic fruits. But you always have to see how much money you have at your disposal.”
**Nutritional improvement wishes and strategies**		
	Eating healthier	Jana: “I often wish that I ate healthier.”
**Test app experiences**		
	High usage effort through tracking	Daria: “So, I did not continue using the app because it was very time-consuming tracking everything and properly. At the beginning it was a lot of fun, but eventually it got harder, because sometimes you do not think about tracking.”
	Visual feedback is used as consumption orientation and promotes self-control	Tino: “Through the app I've noticed that I do not eat enough vegetables. That's why I bought some cucumbers or tomatoes.”

**Table 2:** Excerpt of identified and selected target group preferences for app characteristics and features.

Study 1		Study 2	Target group preferences for app characteristics and features, based on findings from studies 1 and 2	Accept or reject
Topic	Subtopic	Results		
Mobile phone and app usage	Mobile phone is used for entertainment and when bored (eg, games)	Important app characteristics: Entertainment	Features for use when bored/individual time of usage	Accept^a^
Mobile phone and app usage	Listening to music	—^b^	Music	Reject^c,d^
Test app experiences	Disadvantage: High usage effort through tracking (as a result not everything was tracked)	Important app characteristics: Fast use	Supporting low user effort and fast use	Accept^a^
Test app experiences	Advantage: Test-app use for comparison of visual feedback with others	Favorite apps are mostly communications apps	Social comparison	Accept^a^
Test app experiences	Improvement suggestion: More feedback through additional evaluation charts	—	Different evaluation charts	Reject^c,e^

^a^Maintain suspense and adherence.

^b^Not applicable.

^c^Not relevant for target behavior/not in line with target behavior.

^d^Not affordable as incentive.

^e^Focus shall be kept on portions not on calorie intake.

**Table 3:** Intervention functions with capability-opportunity-motivation-behavior components and behavior change techniques (including evidence of effectiveness and target group preferences).

Intervention functions	BCTs^a^ with evidence from literature	Target group preferences
Education	Self-monitoring of behavior [44-46]	Tracking for promoting awareness of eating behavior
Education	Feedback on behavior [47-50]	Tips are motivational (low cost and easy tips)
Education	Information about health consequences [47,51,52]	—^b^
Education	Prompts/cues [53]	Support through reminder
Persuasion	Information about health consequences [47,51,52]	—
Persuasion	Feedback on behavior [47-50]	Tips are motivational (low cost and easy tips)
Persuasion	Verbal persuasion about capability [34]	—
Persuasion	Social comparison [49,54,55]	Social comparison
Incentivization	Feedback on behavior [47-50]	Tips are motivational (low cost and easy tips)
Incentivization	Self-monitoring of behavior [44-46]	Tracking for promoting awareness of eating behavior
Incentivization	Nonspecific incentive/reward (includes positive reinforcement) [49,56,57]	Gamification
Training	Instruction on how to perform a behavior	Tips are motivational (low cost and easy tips)
Training	Feedback on behavior [47-50]	Tips are motivational (low cost and easy tips)
Training	Self-monitoring of behavior [44-46]	Tracking for promoting awareness of eating behavior
Training	Graded tasks [58]	—
Enablement	Action planning^c^ [44]	—
Enablement	Coping planning^c^ [44]	—
Enablement	Goal setting (behavior) [59]	Goal setting
Enablement	Discrepancy between current behavior and goal [46]	Tracking for promoting awareness of eating behavior
Enablement	Self-monitoring of behavior [44-46]	Tracking for promoting awareness of eating behavior
Enablement	Graded tasks [58]	—
Enablement	Social support (unspecified) [55]	—

^a^BCT: behavior change technique.

^b^Not applicable.

^c^Based on the health action process approach [36].

#### Step 6: Developed Preliminary Concept (Prototype I)

This step resulted in the development of the *Challenge to go* (*C2go*) app concept. Multimedia Appendix 4 shows how target group preferences and BCTs were matched and jointly translated into app features.

**Figure 3:**
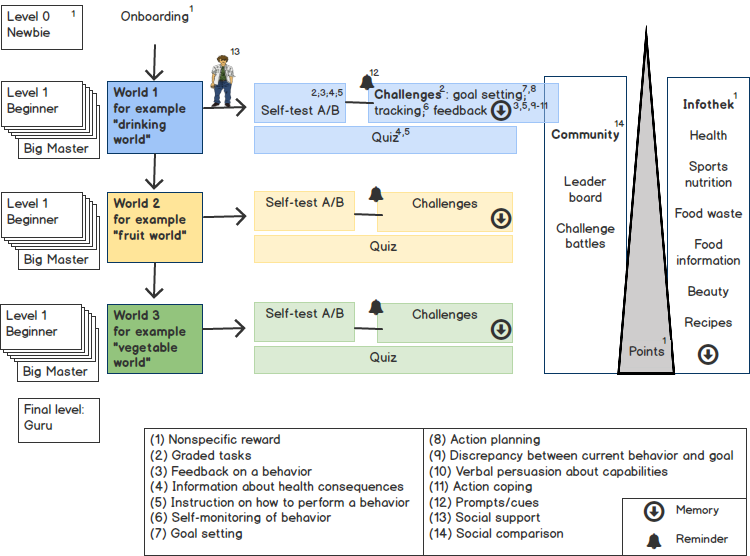
Flowchart of *C2go*.

The following section gives an overview over the concept (Figure 3). After onboarding, the user can choose among 3 worlds: the *drinking*, the *vegetable* **,** or the *fruit world*. In each world, users can accept *challenges* and participate in a *quiz*. To get access to the challenges, users must go through self-tests. Consequently, in a challenge, the user must choose a behavioral goal from a list that he or she tries to achieve, for example, in the fruit world, one portion of fruit per day for 1 week. Challenges can be played alone or against someone else in the community. Different *feedback* is given to motivate and to empower the user to achieve his or her challenge goals, for example, the informative feedback after a lost challenge gives tips on how challenge goals can be reached. Each tip is stored and always accessible for the user. For further support, *reminders* can be set. Through different activities users earn *points* and achieve *levels*. The points open access to the *Infothek*, where users can choose content that is of their interest. The content received from the *Infothek* is stored and always accessible. A leaderboard compares user scores in the *community*, which is made up by other app users. Through passing challenges, users ascend in levels all the way up to *Big Master*. After completing a world, the next world can be selected. If the user has reached the highest level in every world, he or she becomes a *Guru*. Through the whole app, users are guided by an *avatar*.

### Phase III: Evaluation

#### Step 7: Expert Evaluation

Data from expert evaluation and rating results suggested changes for the following app features: worlds, challenges, feedback, *Infothek*, and quiz. Furthermore, the issue of usage motivation was discussed (Table 4).

### Step 8: Final Intervention (Prototype II)

The following sections describe the app features of Prototype II in detail. Further information regarding interventions functions, BCTs, and COM-B components are given in Multimedia Appendix 4.

### Onboarding

Onboarding, that is, the way a user is introduced into the app content, is important to motivate the user for adherence [33]. As such, the app starts with an introductory question (Textbox 1).

The introductory question is used to make users curious and motivate them to use the app through connecting his or her personal aim with app usage and selecting a question that can only be answered correctly, so that the user cannot lose and does not become demotivated [33]. Depending on the answer, a progress bar representing the guru status is implemented, and either titled *Health Guru*, *Wellbeing Guru*, or *Performance Guru*. A user reaches the *Guru* level after succeeding in all 3 worlds.

Consequently, a tutorial gives a brief overview of the app and how to use the app [32,33]. Afterwards, profile settings allow enhancement and customization of the app [33] and are associated with the selection of the avatar in terms of age and sex.

### Self-Test

Each world starts with a self-test, which comprises 2 parts. The first part is a 24-hour dietary recall (eg, vegetables in the vegetable world). Results of this recall are used to divide users into HAPA stages—either intender, if dietary guidelines are not met, or actor, if guidelines are met [19]. The second part is a quiz, which serves as an introduction into the world concept and challenge rules and basics of nutrition education. The answer of each question is formulated depending on the HAPA stage of the user [36] and personal app aim (Textbox 2 for an example), which are stated by the user. After completing both parts of the self-tests, the user gets access to the challenges.

Dissolution: Drinking (at least) 1.5 L per day of sugar-free drinks is good for you and helps you to get closer to your goal: to live healthier.

**Table 4:** Concept changes after expert evaluation.

App feature/characteristic	Prototype I	Prototype II
Challenges	Strict defined rules to reach goals	Easing the rules: More jokers for the users of the drinking world in the *Big Master* level
Feedback	Visual feedback	More visual feedback, for example, a smiling avatar for rewarding consumption of water
Feedback	Informative feedback (tips)	Optional extra button for more information, the user can choose if he/she wants more information concerning feedback
Feedback	—^a^	Reflective questions in feedback, for example, *What are your best tips for a friend to encourage him to drink more water?*
Feedback	—	Goal-orientated feedback: Asking an introductory question, linking the tips to the answer and thereby to the user’s goal
Usage motivation of app	Feedback development: Focus on preintender and intender	Informative feedback focuses on intenders only, preintenders are not considered. This decision was supported by literature, as health apps are more likely used by health-conscious people [31]
Worlds	—	Bonus worlds: Might be possible with an update of the app (menu button *bonus worlds*)
Worlds	Worlds are started one after the other	The worlds can be played simultaneously
Infothek	Point-related access or as a present	More frequently access: Through reduction of the distance of the points
Infothek	Information about healthy snacks/drinks	Including more information about healthy snacks and drinks
Infothek	Health, food waste, and beauty are topics	Content ideas for the following topics: (1) *Health* **:** Where does enjoyment of taste start and where does it end (start of addiction); mechanism of resorption of nutrients; *food* *waste*: Shelf life of food, seasonality of fruits and vegetables, environmental pollution; (2) *Beauty*: Cosmetics without any animal testing
Quiz	Answering question with yes/no; assigning answers	Answering questions with drag and drop mechanisms

^a^Not applicable.

**Introductory question.**

Where can Challenge 2 go support YOU the most?

- Live healthier
- Feeling good in my body
- More fitness and performance

**Example of a quiz question from the second part of the self-test. Example for a user whose personal aim is to live healthier.**

How much should we drink at least a day?

- 0.5 L
- 1.5 L
- 1 L
- 3 L

### Challenges

The challenges are implemented for the user to reach self-imposed consumption goals, which can be chosen out of a list, for example, 3 servings of vegetables per day in the vegetable world or 3 portions of unsweetened beverages in the drinking world (level *Adept* in Table 5).

In the drinking world, a sugar mountain builds up additionally, while tracking sugar-sweetened beverages, which users must reduce through answering quiz questions before attacking the next challenge. In addition, in the fruit and vegetable world, it is not only quantity, but also quality, that counts. Users are motivated to eat as many colors as possible, as recommended in other studies [39,60]. In every world, the aim is to get better in behavioral terms from challenge to challenge up to the highest level, called *Big Master*, which meets the target behavior. To pass the challenges, users get support through feedback and tips. Before finishing a world successfully, every user must play the *Big Master* challenge, even if he or she is already carrying out the behavior and finishing the quiz.

### Feedback and Community

Different feedback is implemented in the *C2go* app: visual, informative, motivational, evaluative, and competitive. An avatar, which is intended as social support [36], gives some visual and all informative, motivational, and evaluative feedback. The avatar rewards user’s desirable behavior with a facial expression (smiling face). Other visual feedback is given through progress bars for different features (self-test, challenges, quiz, and overall progress in app that represents the *Guru Status*) and a graph for an actual versus target feedback for the target behavior. Informative feedback contains messages relevant for intenders (based on HAPA [44]) by encouraging action coping (overcoming barriers, including reflexive questions) and action planning (when, how, where implementing behavior, including instructions on how to perform a behavior) to close the gap between intention and actual behavior [36,39]. Motivational feedback is given through encouraging messages that should sustain intention and self-efficacy [34,48]. Similarly, self-efficacy will be boosted by evaluative feedback [34,39]. When creating feedback, care was taken that this was formulated in a positive way [32,48] and using colloquial language [39]. For examples, refer to Table 6. Furthermore, competitive feedback is given through a leaderboard in the *community* [34]. In addition, challenges can be played alone or against other app user in the community to promote motivation for behavior change through social comparison and competition [54,61]. The community consists of all app users.

### Reminder

Reminders are push notifications and can be set to support users on the way to meeting their goals. Furthermore, they function as a re-engagement tool [53]. Types of different reminders are presented in Table 7. Every reminder can be switched off or on.

**Table 5:** Levels in the drinking world of the app.

Phase	Level
After selecting first world	Beginner
After completing self-test B	Climber
After the first challenge (24-hour-Challenge)	Adept
3 portion challenge	Adept pro
4 portion challenge	Expert
5 portion challenge	Expert pro
6 portion challenge	Master
6 portion challenge + no sugary drinks	Big Master

**Table 6:** Feedback examples.

Feedback type	Time point	Example
Motivational	During challenges	*Great first 7 days!*
Evaluative	After a challenge	*Try again: the next level is already waiting for you!*
Informative	After a challenge	*Drink a portion of unsweetened beverage with each meal, for example, (mineral) water or herbal/fruit tea (warm or cold)*

**Table 7:** App reminders and time points.

Reminder	Time point
Consumption of target behavior	From 9 am to 9 pm, every 3 hours
Tracking	9 pm
Start of challenge	9 am
End of challenge day	9 pm, if necessary next day 7 am und 12 pm
End of a challenge	Immediately, if necessary 36 hours and 48 hours later
Infothek	If access has been granted

### Quiz

In terms of content, the quiz aims to provide knowledge about the health-related value of each target behavior and the national intake recommendations. To this end, each world has its own quiz, for example, the fruit world quiz contains questions regarding fruit intake recommendations and associated health benefits and risks (Textbox 3 for an example). All questions must be answered correctly to complete the respective world. Wrongly answered questions will be repeated later.

**Example of quiz question.**

How long can humans survive without liquid intake?

- 2-4 days
- 1 week
- 1 day
- 50 days

Dissolution: We (humans) can live without solid food for more than a month, but without drinking we die after 2-4 days!

### Infothek

The *Infothek* is an information section where users get access to interesting information regarding 6 nutrition-related topics, which were derived from study results described above: health, food information, beauty, sports and food, food waste, and recipes. Users get access at certain scores to get motivated to app usage and reward it. Regardless of the score, once a week access to the *Infothek* is given to re-engage the user. Information is predominantly in written form (short messages). Also, short videos and podcasts are intended.

### Gamification Approach

The *C2go* app implements severely playful elements, such as points, levels, leaderboard, challenges, onboarding, feedback, progress bars and customization, to engage users.

## Discussion

### Overview

Mobile phone-based interventions are increasingly used to promote a healthy lifestyle [30,62-65]. In this study, a mobile phone app was considered as a very acceptable tool for the delivery of a nutrition intervention for adolescents and young adults, as mobile phone usage is widespread across all education and income levels [66]. Furthermore, adolescents and young adults are at a stage of life where their own lifestyles, including *eating styles*, are developed and established. The empirical study results described above confirm the general openness for, and interest of adolescents and young adults in, a dietary mobile app. Next, other digital health interventions were rated as helpful and satisfactory by adolescents and young adults [57,67,68] and other age groups [69,70]. Studies by others have demonstrated that mobile phone apps are widely accepted by users for intervention delivery in the field of healthy eating [30,62,71]. Despite the growing interest in mHealth research, the development of a theory-driven app is not well described in the scientific literature [30]. Furthermore, to our knowledge, no dietary mobile app has been designed to meet the needs and interests of adolescents and young adults in Germany. Our study provides a step-by-step description of how evidence (eg, from empirical studies with the target group) and theory can be translated systematically into an app concept in the field of mHealth for contributing to the prevention of NCDs.

### Systematic Design Process

The concept for the C2go app was designed along the BCW and based on the input of the target group as well as the literature analysis. The BCW has been used by other researchers to guide the development of mHealth interventions [30,72].

Effective interventions need theory guidance [36,73]: Using a framework helps design interventions systematically, deriving factors that need to be changed and avoiding intervention development based on personal experiences, favorite theories, or superficial analyses [40,73,74]. Besides this, theory-based interventions help to understand which, and how, techniques are effective, and results can be used to optimize theoretical concepts. Furthermore, using theory in research is also helpful for the communication between researches and disciplines [73]. Often, the use of underlying theories and concepts in intervention trials is not well described. Theories are only mentioned as frameworks but descriptions of how they were integrated into the scientific design process are often lacking [74]. In this study, the use of the BCW as a framework permitted the systematic and comprehensive design process, which was underpinned by a model of behavior change and a behavioral diagnosis of the target behavior, before starting the design process. However, it is necessary to expand the BCW regarding the derivation of empirical study results and its translation into BCTs and, finally, into mHealth app features [30]. This could minimize the influence of individual expertise, creativity, and reasonable decision of scientists on which features should actually be implemented in the app [30].

A major strength of this study was the examination of the behavior, interests, needs, and values of the target group, because digital interventions are most engaging when they are matched to the target group’s characteristics, needs, expectations, and skills [75]. Following this, other studies revealed that involving the target group throughout all phases of intervention development is important to make it more relevant to their life [30,63,68]. Our study therefore aimed at focusing on adults from disadvantaged backgrounds. Therefore, attempts were made to recruit study participants in (public) places with lower educational background in particular, for example, vocational school.

In total, 3 target behaviors were initially chosen, because concentrating on many or unspecified target behaviors (eg, whole nutritional intake) is assumed to be less effective than considering only a few and specified target behaviors (but then intensely) [76]. The selection of the target behaviors was indirectly confirmed by the target group, as beverages, fruit, and vegetables were voted as easy-to-track food. A further advantage of choosing the 3 target behaviors is that they focus on *promotin* g individual behaviors, for example*, more* fruits, instead of forbidding food (eg, *eat only 1 piece of candy per day*).

Altogether, study results along with literature searches were used to support the behavioral diagnosis, the identification of intervention functions, and BCTs. The behavioral diagnosis revealed that certain capabilities (eg, psychological capability: awareness of consumption), opportunities, and motivational aspects are needed to establish healthy eating habits. Furthermore, study results together with evidence from the scientific literature were used to identify 5 intervention functions and 14 BCTs. The latter were translated into features of the *C2go* app.

### Final Intervention: The Challenge to Go App

The *C2go* app concept targets improved drinking habits as well as increased fruit and vegetable consumption among adolescents and young adults. To this end, users choose from 3 worlds: the drinking, the vegetable, or the fruit world. A core feature of the *C2go* app concept is the use of challenges. These consist of goal-setting and self-monitoring for target behavior. Both techniques were requested by participants and are supported by evidence from the literature [36,45,58,77,78]. Focusing on BCTs for effective behavior change interventions is important. Nevertheless, considering determinants of engagement is also crucial [75]. Therefore, the *C2go* app concept implements various game elements and process motivators, which reward the process of behavior change. Examples are points, levels for status gain, rankings, or engagement loops through challenges aiming at edutainment and loyalty [33,34]. Gamification approaches can provide motivation in settings where information only is not sufficient to bring about change [34]. Various other mHealth interventions used gamification for promoting user engagement successfully [30,43,63]. Concentrating on process motivators instead of long-term, logical outcome motivators (eg, prevention of NCDs) is proposed to influence self-efficacy for behavior change positively and more effectively [79].

The individual choice of worlds and, inter alia, the setting of individual goals support customization [33] to satisfy different needs and motivation for app use (engagement). Reminders were also implemented to increase user engagement [53]. Furthermore, the implementation of an avatar will improve user engagement and acceptance [80].

Different feedback was implemented to motivate app usage [54] and behavior change. Implemented informative feedback targets intenders. This serves to boost self-efficacy, thereby helping to overcome barriers and to achieve target behavior [36,51]. Visual feedback through progress bars was used to replace possible missing intrinsic motivation for behavior change [34]. Evaluative feedback, such as *Congratulations* if challenges are passed or encouraging feedback if challenges are not passed, were implemented to increase self-efficacy [43]. Motivating messages serve to increase self-efficacy in encouraging the idea that skills for succeeding are available [34]. Evaluative, informative, and motivating feedback is given through an avatar that was implemented for identification and positive learning effects [34]. When formulating this feedback, it was important to select positive language to increase the self-efficacy and satisfaction of the user [48]. Competitive feedback comes from the community and the leaderboard [34,43].

### Limitations and Future Research

Several limitations must be considered when interpreting the findings of the present approach. First, the design, development, and implementation of mHealth concepts take time. Consequently, by the time of implementation, technology and target group interests may have evolved [30]. Second, regarding the target behaviors, the app focused on drinking and fruit and vegetable consumption only. Other food groups and nutrition behaviors (eg, snacking) could be targeted in the app at a later stage of development, along with physical activity. This provides opportunities for future research and extension of the app. Third, the participants who assisted in the app development were only from 1 region in Germany, and they may have had a bigger interest in nutrition or apps as nonparticipants. Future investigation should include a more diverse group of participants.

The next step is the validation of the *C2go* app concept to demonstrate its impact on drinking and fruit and vegetable consumption, as well as its usability in a controlled intervention trial. Moreover, financial opportunities for sustainable maintenance possibilities of scientific applications must be investigated.

### Conclusions

*C2go* is a theory-based and target group–adapted mobile intervention that was systematically developed using the BCW. *C2go* aims to improve drinking habits and the consumption of vegetables and fruit among adolescents and young adults, especially from disadvantaged backgrounds, using a gamification approach.

## Appendix

Multimedia Appendix 1 Additional information on studies 1 and 2.

Multimedia Appendix 2 Behavioral diagnosis to derive what needs to be changed to achieve the target behavior and estimation of feasibility in a dietary mobile app.

Multimedia Appendix 3 Results of studies 1 and 2.

Multimedia Appendix 4 Bringing together behavior change techniques and target group preferences for derivation of app features.

## Abbreviations

APEASE: affordable, practicable, effective, acceptable, side-effects, equity
BCT: behavior change technique
BCW: behavior change wheel
C2go: Challenge to go
COM-B: capability-opportunity-motivation-behavior
HAPA: health action process approach
mHealth: mobile health
NCD: noncommunicable disease
