# Supplementary material for: An App to Improve Eating Habits of Adolescents and Young Adults (Challenge to Go): Systematic Development of a Theory-Based and Target Group–Adapted Mobile App Intervention
Source: JMIR Mhealth Uhealth. 2019 Aug 12;7(8):e11575. doi: 10.2196/11575 (PMC6709564; doi:10.2196/11575)
Supplement: Multimedia Appendix 1 [file mhealth_v7i8e11575_app1.pdf]

Multimedia Appendix 1. Additional information on studies 1 and 2

Study 1

Recruitment and compensation: Recruitment took place via gatekeeper. The gatekeepers were staff from youth centres, schools, vocational preparation interventions, social-education youth projects, institutions, youth church communities and two nutritionists. A total of 54 people from different institutions were contacted by mail, by phone or via Facebook. Due to a low response rate, incentives were increased from a raffle to an Amazon voucher of 25 Euros. A total of 24 people showed interest and received study information. Finally, eleven participants completed the study. The early drop-out reasons were: Problems in the clarification of the signature authorization (n= 1), did not show up the interview (n= 1), and most often no further feedback (n= 11). An inclusion criterion was: age between 14-25 years; exclusion criteria were: pregnancy and breastfeeding, or chronic diseases.

Interview guide (freely translated):

1. What do you do in your spare time?
2. On which occasions do you use your mobile phone?
3. Which apps do you prefer to use?
4. Have you ever downloaded an app in the field of nutrition?
5. You tried the “Was ich esse” app for a week: When did you use it?
6. What did you like?
7. What didn’t you like?
8. Which improvements would you suggest?
9. What did you eat yesterday?
10. When you eat, what is important to you?
11. Do you go food shopping yourself?
12. Do you cook yourself?
13. When you look at the pictures (pictures of a supermarket, kitchen, salad, French fries, jogger, chicken) what do you have to think about at first?
14. What does "healthy food" mean to you?
15. What do you think, what brings you a "healthy diet"?
16. How do you like your own diet?
17. Would you possibly change anything in your diet?
18. What could support you? What is stopping you?

Test app “Was ich esse“

The “*Was ich esse*” (in English: “What I eat”) app was used as test app. It can be downloaded for free in the Google Playstore or the Apple Store. Users can track what they have eaten throughout the day. Tracking is done with the help of serving sizes (1 serving = 1 hand). The result is visualized in a food pyramid (see **Error! Reference source not found.**).

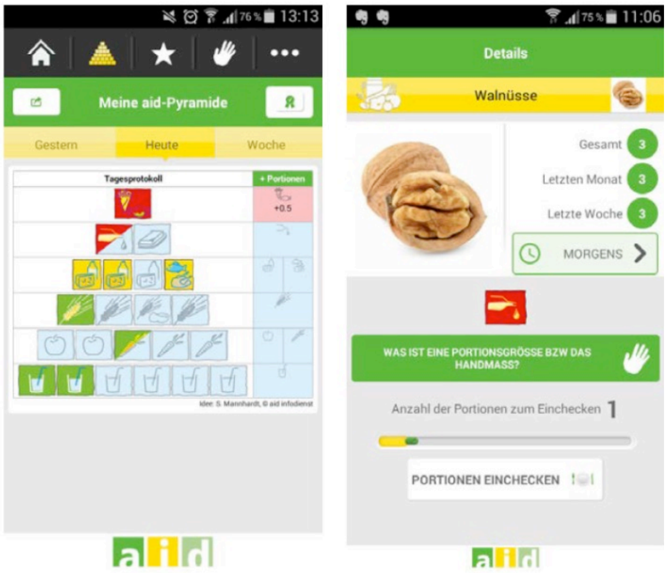

Figure 1 Test app „Was ich esse” (left: The food pyramid; right: Tracking with serving sizes).

Study 2

Recruitment: The recruitment of participants took place from May to September 2016 through gatekeepers. Gatekeepers were teachers, social workers/educators or other persons that work in schools, youth services, social education/youth services or pre-vocational preparation classes. The gatekeepers were contacted by email. Among the contacted institutions were 13 schools, four pre-vocational interventions, five youth centres and 24 social-educational (youth) projects and institutions. In addition, students who carried out their seminar work in cooperation with the University of Jena were asked personally or by e-mail. Collaboration was possible with five institutions. An inclusion criterion was: age of 14-25 years. The gatekeepers or the author managed the distribution and collection of the statements of consents and questionnaires. Every participant completed the questionnaire on a voluntary basis.

Compensation: Among all participants, three Amazon (25€) vouchers were raffled.

Information on the questions in the questionnaire:

- One choice question: Mobile operating system, mobile phone rate, sex, graduation, current school/occupation, citizenship
- Open question: Favorite apps, name of used dietary mobile app(s), age
- Three-point rating scale question: Importance of different app characteristics (e.g. importance of customizability, sharing of videos/photos, no costs for app use); nutritional interests (e.g. sports nutrition, health, food waste); nutritional values (e.g. freshness of food, self-cooked meals, little time-consuming)
