# Supplementary material for: An App to Improve Eating Habits of Adolescents and Young Adults (Challenge to Go): Systematic Development of a Theory-Based and Target Group–Adapted Mobile App Intervention
Source: JMIR Mhealth Uhealth. 2019 Aug 12;7(8):e11575. doi: 10.2196/11575 (PMC6709564; doi:10.2196/11575)
Supplement: Multimedia Appendix 2 [file mhealth_v7i8e11575_app2.pdf]

Multimedia Appendix 2. Behavioral diagnosis to derive what needs to be changed to achieve the target behavior and estimation of feasibility in a dietary mobile app

**COM-B**	**What is required to achieve changes of the target behavior?**	**Need for change?** *Quotes from participants of study 1*	**Feasible?** in a dietary mobile app
**Psychological capability**	Knowledge about recommendations (5 a day, > 1.5 liter sugar free drinks)	Yes [1]	Yes
	Knowledge about health promoting effect of fruits and vegetables, drinks and risk perception	Yes<br>*Leon: "I think I've had too little nutritional problems so far that I say I have to change a lot."*	Yes
	Knowledge about how to realize recommendations (including knowledge about fruits/ vegetables, preparation and consumption, creativity, inspiration, planning skills, alternatives	Yes<br>*Caro: "Healthy recipes, I think that would be good."*	Yes
	Knowledge about behavior change techniques (e.g. goal setting, self-control)	Yes<br>*Caro: "Well, I know that I eat and drink way too much unhealthy drinks. And I always try to change that, but that's so hard for me."*	Yes
	Awareness of consumption and comparison with recommendations	Yes<br>*Daria: "Yeah, I noticed that I eat fewer vegetables than I thought (laughs), and then I thought about it, ok, when can I eat vegetables or fruit again today?"*	Yes
	Self-confidence	Yes<br>*Interviewer: "How satisfied are you with your diet?"*<br>*Caro: "(sighs) not at all. I know that I eat and drink way too much unhealthy. And I always try to change that, but that's so hard for me."*	Yes
**Physical capability**	Have physical skills to buy, prepare or eat/drink fruits and vegetables, drinks	No [2]	-
**Reflective motivation**	Health is a value and trust in health promotion by eating 5 a day and drinking > 1,5l sugar-free beverages	Yes<br>*Leon: "I think I've had too little nutritional problems so far that I say I have to change a lot now."*	Yes
	Knowledge, that realizing 5 a day will require improved skills	Yes [3]	Yes
	Optimistic attitude/openness for change, e.g. by connecting healthy eating with individual goals, e.g. fitness, concentration, illness prevention, strengthen immune system, weight lost, well-being, saturation	Yes<br>*Leon: "I think I've had too little nutritional problems so far that I say I have to change a lot now."*	Yes
	Preferences for healthy foods (demotivator: waiver)	Yes<br>*Jenny: "Yeah, I do not want to give up eating something out of duty."*	Yes
**Automatic motivation**	5 a day and drinking recommendations as established routine and habit	Yes [3]	Yes

**Social opportunity**	Social origin/group identity	Yes [4]	No[a]
	Independence	Yes<br>*Maria: "My mom loves these minute steaks with noodles, so I always have to make tomato sauce, but I'm fed up with noodles (laughs). And sometimes I think, why can't I eat potatoes?"*	No[a]
	Support from family/friends	Yes<br>*Maria: "To make it easier for me to change my eating habits my parents would have to help me, e.g. by telling me to put the cake away and take an apple instead."*	No[a]
**Physical opportunity**	Financial resources	Yes [5]<br>*Jenny: "I go shopping almost every day, with my friend, we buy relatively little and always the cheapest, because I have to pay for it myself and we buy a lot of ready-to-eat products."*	Yes[b]
	Availability: Offer in supermarkets	No [6–8]	-
	Offers in (school)canteens and restaurants	Yes<br>*Interviewer: In the cafeteria?*<br>*Caro: Yes. But I do not think it's good that they offer few fruits and vegetables."*	No[a]
	Availability at home, school, work	Yes<br>*Emil: "I do not go shopping alone and I rarely help my parents or grandparents shopping"*	No[a]
	Time	Yes [5]<br>*Tino: "Well, with warm meals, the app can't help you a lot, because it cannot give you the time you need to cook something (laughs)."*	Yes [c]

[a] No influence through an app; [b] Though no direct influence through an app, tips for non-expansive buying could be given; [c] Though no direct influence through an app, tips for fast preparation could be given

References

1. Bechthold A, Wendt I, Laubach B, Mayerböck C, Oberritter H, Nöthlings U. Consumers' awareness of food-based dietary guidelines in Germany: Results of a representative survey. Ernährungs-Umschau 2017(7):112-119. doi:10.4455/eu.2017.02
2. Remijn L, van den Engel-Hoek L, Satink T, Swart BJM de, Nijhuis-van der Sanden MWG. "Everyone sees you sitting there struggling with your food": Experiences of adolescents and young adults with cerebral palsy. Disabil Rehabil 2018:1-8. PMID:29558834
3. Michie S, Atkins L, West R. The behaviour change wheel: A guide to designing interventions. Croydon: Silverback Publishing; 2014. ISBN:978-1-291-84605-8.
4. Schienkiewitz A-K, Brettschneider SD, Schaffrath Rosario A. Übergewicht und Adipositas im Kindes- und Jugendalter in Deutschland – Querschnittergebnisse aus KiGGS Welle 2 und Trends. Journal of Health Monitoring 2018;3(1):16-23. doi:10.17886/RKI-GBE-2018-005
5. Andajani-Sutjahjo S, Ball K, Warren N, Inglis V, Crawford D. Perceived personal, social and environmental barriers to weight maintenance among young women: A community survey. Int J Behav Nutr Phys Act 2004;1(1):15. doi:10.1186/1479-5868-1-15
6. EHI. Durchschnittliche Artikelzahl der großen Supermärkte bei Obst und Gemüse in den Jahren 2000, 2008, 2013 und 2015 2018.
7. EHI. Durchschnittliche Artikelzahl der Lebensmittel-Discounter im Bereich Frischwaren in den Jahren 2007, 2012 und 2015 nach Warengruppen 2018.
8. Statista. Anzahl der Filialen im Lebensmitteleinzelhandel pro eine Million Einwohner in Europa nach ausgewählten Ländern im Jahr 2015 2018.
