# Supplementary material for: An App to Improve Eating Habits of Adolescents and Young Adults (Challenge to Go): Systematic Development of a Theory-Based and Target Group–Adapted Mobile App Intervention
Source: JMIR Mhealth Uhealth. 2019 Aug 12;7(8):e11575. doi: 10.2196/11575 (PMC6709564; doi:10.2196/11575)
Supplement: Multimedia Appendix 3 [file mhealth_v7i8e11575_app3.pdf]

Multimedia Appendix 3. Results of studies 1 and 2

Results study 1 (n= 11)

Nutritional habits

- Food shopping: Yes or no
- Breakfast: Either at home, on the road, at school or not at all (lack of hunger)
- Lunch: Self-made sandwiches, self-cooked meals, food of the cafeteria/canteen or not at all
- Dinner: Something warm or cold
- Snacks: Yes (fruits, cake, ice cream, biscuits) or no
- Beverages: Much or little, coffee or not
- Influences on nutrition habits: Family (meals), friends, offer in canteen, time, cooking skills, preferences, financial opportunities, hunger, life situation (see Change in nutrition behavior) recipe apps and websites, stress in school or university, "everyday wisdoms", missing self-confidence
- Change in nutrition behavior (intrapersonal): Pregnancy vs. non-pregnancy, fasting time vs. non-fasting time, weekdays vs. vacation/weekend, visit to the grandma's house vs. eating at home or high school time vs. time after high school

Mobile phone and app usage

- Mobile phone is used for entertainment:
  Playing games, e.g. Color Switch, Doodle Jump, Quizduell, Pokémon Go, Block! Hexa, card games, looking YouTube Videos, listening to music
- Mobile phone is used when bored
- Mobile phone is used for communication with family and friends:
  Phone calls, messages via WhatsApp, Facebook, iMessenger, Instagram, SMS, emails, sharing of pictures
- Mobile phone is used as tool for every day relief:
  Google maps, menu app for canteen menu, as clock, dietary mobile app for weight management, camera functions, internet research, app that helps learning bible verses by heart, tuner for guitar, no costs for some apps
- Memory of mobile phone limits app use

Nutritional improvement wishes and strategies

**Nutritional improvement wishes**	**Nutritional support strategies**
Regular warm lunches; e.g. lunch at school	More time to cook; support from friends and family
Eating healthier	Support from friends and family; more self-control/motivation; more self-confidence; support and motivation by dietary mobile app; alternative food ideas; offer of fruits and vegetables in the school canteen
Eating fewer incompatible foods	Shopping aid for finding lactose-free products quickly; broader offer of lactose-free products in supermarkets; financial support; more self-control/motivation
Less sweets	alternative food ideas; support by dietary mobile app that displays consumption

Smaller portion sizes	Tracking of consumption
More fruit and vegetables	Support through dietary mobile app
Drinking better	Support through dietary mobile app
Learn to cook	Practice cooking
Maintain weight	Appropriate eating environment; more self-control/motivation
Lose Weight	Support from friends and family; alternative food ideas; support through dietary mobile app
Cooking more often freshly	Cooking inspiration (e.g. healthy recipes)

Nutritional values

- Food shopping: Organic and regional food
- Food shopping: Unpacked food
- Food shopping: Buying meat from animal welfare
- Food shopping: Appealing appearance of mid-life/packaging
- Food shopping: Natural food ingredients
- Food shopping: Fresh with fruits/vegetables
- Food shopping: Spending on food should be kept low
- Food consumption: Satisfy physiological needs
- Food consumption: Taste and preferences
- Food consumption: Compatibility of food
- Food consumption: Pleasure food instead of abandonment
- Food consumption: To try something new
- Food consumption: Uncomplicated, easy-going
- Food consumption: Varied, balanced diet
- Food consumption: Less stress in school
- Food consumption: Support from family
- Food consumption: Appropriate eating environment
- Food consumption: Follow nutritional guidelines
- Food consumption: Structuring of daily eating
- Cooking: Little effort when cooking (e.g. in the evening after work)
- Cooking: Conviviality when cooking
- Cooking: Cooking for fun, challenge and good feelings
- Cooking: Independent buying/eating decisions
- Cooking: Cooking stands for independency
- Nutritional goals: Building muscle through nutrition
- Nutritional goals: Energy/concentration
- Nutritional goals: Fitness, performance and concentration through a healthy diet
- Nutritional goals: Disease prevention
- Nutritional goals: Strengthen your immune system
- Nutritional goals: Lose body weight
- Nutritional goals: High enough body weight

Understanding of health

**What is healthy?**	**What is unhealthy?**
Sports	Frozen products

Moving in fresh air	Snacks
Conscious eating and taking time	Cola
Rice/noodles and others grain products	Crisps
Fresh food	Fast food chains
Beverages: Drinking enough and water	Snacking: Sweet and salty snacks and too much
Regularly eating	Ready meals, e.g. Pizza
Hearty food with a little sweet for nutrients	Sweets, e.g. gummy bear
Freshly cooked food	Nutella
Balanced eating	French fries
Vegetables and fruits	Meat: Too much and e.g. pork meat
High in fiber	Greasy food
Less fat	Unconscious eating
Self-cooking for the baby better than a glass	You should not eat so much of unhealthy foods
Diet and exercise together are part of a healthy lifestyle	Unhealthy fast food is allowed from time to time
A healthy diet is a challenge	
Health level of the nutrition behavior is from day to day different	
Healthy foods are not consumed when they do not taste good	

Assessment of estimated effect of a healthy lifestyle

- Healthy diet: Nutrient supply; disease prevention/strengthening immune system; no bloating; does the body good: Conserves and cares; satiety; fitness; performance; concentration; weight loss; self-cooked; healthy diet (participant found no other explanation)
- Exercise: For well-being

Assessment of the estimated effect of an unhealthy lifestyle

- Unhealthy eating: No need for the body; does not do well and hurts your health; makes you fat; influences the behavior; impact on body in digestion; low importance and risk perception regarding health consequences

Test app experiences

Advantages	Disadvantages	Improvement suggestions
Simple tracking with serving sizes for certain foods: Fruits, water	Estimation of portion sizes: Not easy	Serving sizes: More accurate definition and description
App use influenced nutrition behavior positively: More drinking, fruits and vegetables, less sweets, less lactose-free products		
App can be used to track consumption, instead of manually logging consumption to control portion size		

App usable to kill boredom (e.g. visual feedback seen)		
Searching and tracking food with a drop-down list facilitates tracking	Search problems	Search system: By voice input<br><br>Search system: Category-based
Setting goals: Personalizing and<br>motivating	Setting goals by users make sense?	Pretend goals through dietary mobile app, instead of by users
Great food database: Facilitates tracking	Food database: Not complete	Expand food database
Menu item for information on serving sizes are helpful	High usage effort through tracking (as result not everything was tracked): Tedious and time-consuming<br><br>Tracking not easy to think of	
	Tracking in day sections (e.g. breakfast or dinner)	
Comparison of visual feedback with other users (= motivating for attaining goals)	App use in the beginning exciting, but fast boring	
Adding missing food in the food database	Food group beverages: No sugar sweetened beverages count to the liquid supply	
Collection badges promotes self-confidence	Badges: Meaning incomprehensible	
(Daily and weekly) visual feedback (actual vs. target) promotes awareness of consumption		Additional evaluation charts, e.g. calorie needs consumption chart
(Daily and weekly) visual feedback (actual vs. target) is used as consumption orientation and promotes self-control		
		Nutrient-related information to foods, evaluation of food
Reminders: Individually and adjustable		Expand reminders
		Healthy recipes
		Shopping tips
	Lack of personalization	Personalization (e.g. age, height, weight)
Favorites: Facilitate tracking from too much consumed food		
Pictures of serving sizes: Orientation and inspiration for consumption ideas		
Visual design		

Usability = easy and uncomplicated		

Results study 2 (n= 210)

Operating system

Table 1 Operating system (n= 210)

Operating system	Number of participants
Android	160
iOS	38
Android and iOs	2
Windows Phone	6
Other	2
No Mobile phone	2

Mobile phone Tariffs

Table 2 Mobile phone Tariffs (n= 204, no data provided n= 6))

Operating system	Number of participants
Prepaid	41
Flat rate < 500 MB	53
Flat rate > 501 MB	70
Flat rate > 501 MB and others	1
Others	39

Favorite apps

Overall, participants (n= 204, no data provided n= 6) specified 793 apps, including 160 different apps. The following list shows apps that were named three times and more:

1. WhatsApp (n= 186)
2. Facebook (n= 124)
3. Instagram (n= 88)
4. Snapchat (n= 60)
5. Youtube (n= 57)
6. Clash of Clans (n= 12)
7. Facebook Messenger (n= 10)
8. Twitter (n= 9)
9. Clash of Kings (n= 8)
10. DB Navigator (n= 7)
11. Google (n= 7)
12. PokémonGo (n= 7)
13. Lovoo (n= 5)
14. Units (n= 5)
15. Skype (n= 5)
16. Amazon (n= 4)
17. Candy Crush (n= 4)
18. Messenger (n= 3)
19. Spotify (n= 3)
20. Retrica (n= 3)
21. Mp3Player (n= 3)
22. 4 Bilder 1Wort (n= 3)
23. 9gag (n= 3)
24. Musical.ly (n= 3)
25. Deezer Music (n= 3)
26. Hay Day (n= 3)

Experiences with apps in the field of nutrition

54 of the participants have experiences with apps in the field of nutrition (no information provided n= 2). 23 different apps were specified by 26 participants.

Table 3 Apps in the field of nutrition (n= 26)

Category	Apps
**Food waste**	Beste Reste (n= 1); Zu gut für die Tonne (n= 1)
**Fast food**	Burger King (n= 1)
**Recipes**	Chefkoch (n= 25); Kochbar (n= 1); Kptn Cook (n= 1)
**Weight control and diet**	Noom Coach (n= 1); Weight Watchers (n= 1); Diätplan (n= 1); Ernährungsplan (n= 1) ; FatSecret (n= 1) ; FDDB-Extender (Kalorien-Kalender) (n= 1) ; Was ich esse (n= 1); Yazio (n= 4)
**Food ingredients**	Codecheck (n= 1)
**Preparation help**	Eier Uhr (n= 1)
**Ordering service**	Lieferheld (n= 3)
**Nutrition and fitness**	Fit-Trio (n= 1); Fitness Coach (n= 1) ; Fitness Lounge (n= 1) ; S-Health (n= 4) ; MyFitnessPal (n= 1)
**Social media**	Pinterest (n= 1)

Importance of app characteristics

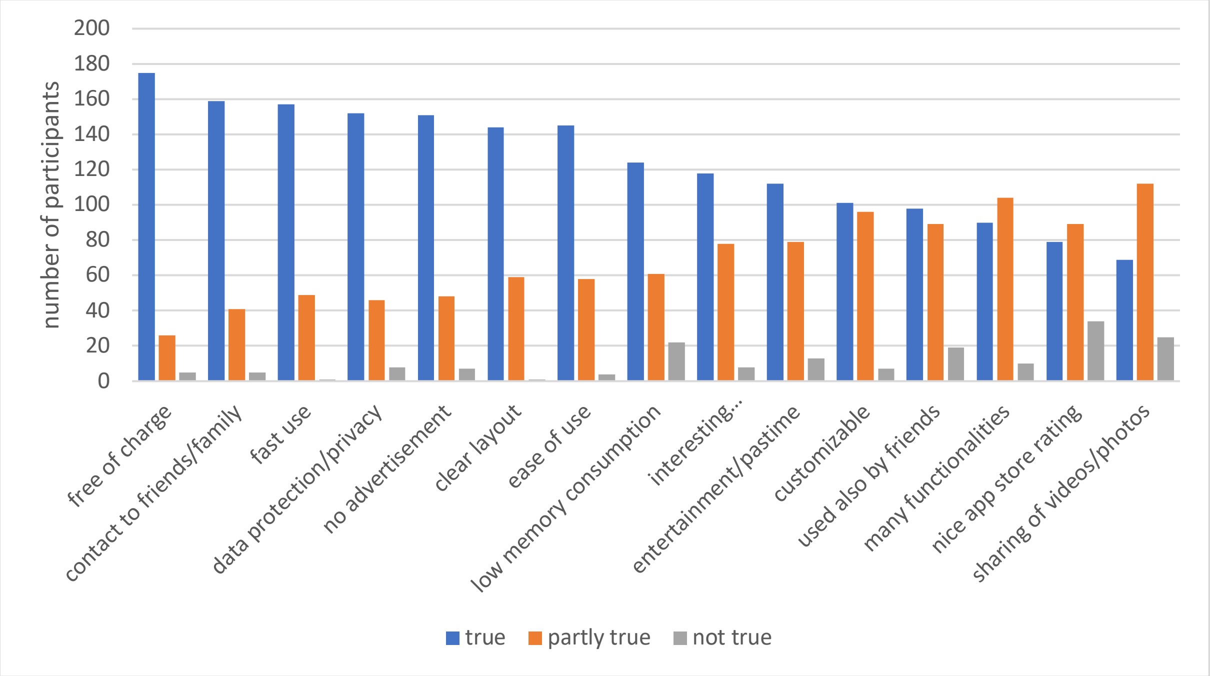

Figure 1 Importance of app characteristics

Table 4 Importance of app characteristics

	True	Partly true	Not true	No data
**Free of charge**	175	26	5	4
**Contact to friends/family**	159	41	5	5
**Fast use**	157	49	1	3
**Data protection/privacy**	152	46	8	4
**No advertisement**	151	48	7	4

**Clear layout**	144	59	1	6
**Ease of use**	145	58	4	3
**Low memory consumption**	124	61	22	3
**Interesting info/functionalities**	118	78	8	6
**Entertainment/pastime**	112	79	13	6
**Customizable**	101	96	7	6
**Used also by friends**	98	89	19	4
**Many functionalities**	90	104	10	6
**Nice app store rating**	79	89	34	8
**Sharing of videos/photos**	69	112	25	4

Interesting topics in the field of nutrition

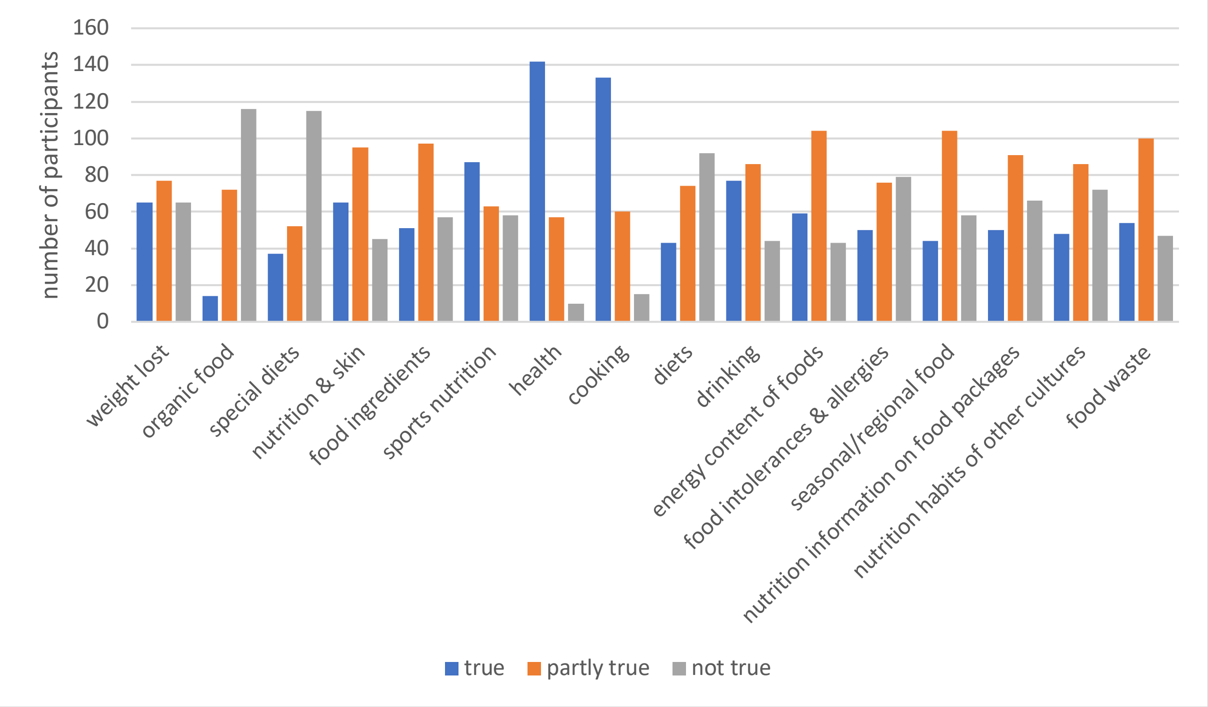

Figure 2 Interesting topics in the field of nutrition

Table 5 Interesting topics in the field of nutrition

	True	Partly true	Not true	No data
**Weight lost**	65	77	65	3
**Organic food**	14	72	116	8
**Special diets**	37	52	115	6
**Nutrition and skin**	65	95	45	5
**Food ingredients**	51	97	57	5
**Sports nutrition**	87	63	58	2
**Health**	142	57	10	1
**Cooking**	133	60	15	2
**Diets**	43	74	92	1
**Drinking**	77	86	44	3
**Energy content of foods**	59	104	43	4

**Food intolerances and allergies**	50	76	79	5
**Seasonal/regional food**	44	104	58	4
**Nutrition info on food packages**	50	91	66	3
**Nutrition habits of other cultures**	48	86	72	4
**Food waste**	54	100	47	9

Nutritional values

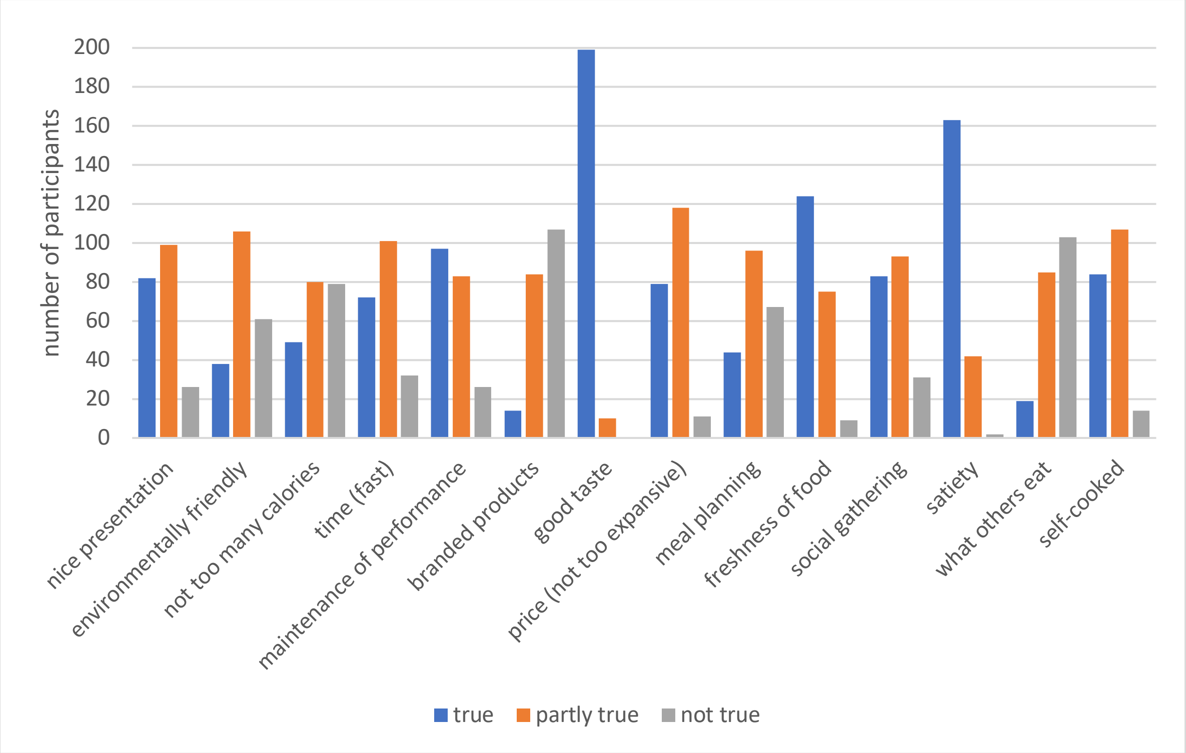

Figure 3 Nutritional value

Table 6 Nutritional values

	True	Partly true	Not true	No data
**Nice presentation**	82	99	26	3
**Environmentally friendly**	38	106	61	5
**Not too many calories**	49	80	79	2
**Time (fast)**	72	101	32	5
**Maintenance of performance**	97	83	26	4
**Branded products**	14	84	107	5
**Good taste**	199	10	0	1
**Price (not too expansive)**	79	118	11	2
**Meal planning**	44	96	67	3
**Freshness of food**	124	75	9	2
**Social gathering**	83	93	31	3
**Satiety**	163	42	2	3
**What others eat**	19	85	103	3
**Self-cooked**	84	107	14	5
