# Supplementary material for: An App to Improve Eating Habits of Adolescents and Young Adults (Challenge to Go): Systematic Development of a Theory-Based and Target Group–Adapted Mobile App Intervention
Source: JMIR Mhealth Uhealth. 2019 Aug 12;7(8):e11575. doi: 10.2196/11575 (PMC6709564; doi:10.2196/11575)
Supplement: Multimedia Appendix 4 [file mhealth_v7i8e11575_app4.pdf]

Multimedia Appendix 4. Bringing together behavior change techniques and target group preferences for derivation of app features

C2go app features	Target group preferences	BCTs	Intervention functions // COM-B components
Onboarding [(a)]	Customization/individual needs, ease of use, instructions of rules	Non-specific reward	Incentivization // Automatic and reflective motivation
Self-test part 1	Tracking for promoting awareness of eating behavior; visual actual vs. target feedback for consumption orientation/awareness and self-control promotion	Self-monitoring	Education, training // Psychological capabilities, reflective motivation
Self-test part 2	Instructions of rules	Information about health consequences; Instruction on how to perform a behavior	Education, training // Psychological capabilities, reflective motivation
Challenges in different worlds	Supporting low user effort and fast use; maintaining suspense; no stiff nutritional program, as intra- and interpersonally different eating behavior, gamification, Customization/individual needs	Graded tasks	Training, empowerment // Psychological capabilities, automatic motivation
Goal setting	Customization/individual needs; goal setting	Goal setting, action planning, action coping	Empowerment // Reflective and automatic motivation
Tracking (one food group only)	Tracking and visual (actual vs. target) feedback for consumption orientation/awareness and self-control promotion; supporting low user effort and fast use, favorites, information on portion sizes, category-based food search, tracking must be simple, tracking with portion sizes, drop-down lists, vast food data base	Self-monitoring (behavior)	Education, training, empowerment // Reflective and automatic motivation; psychological capabilities
Visual feedback (self-test part 1, actual vs. target graph, sugar mountain, color graph)	Features for use when bored/ individual time of usage; tracking and visual (actual vs. target) feedback for consumption orientation/awareness and self-control promotion	Feedback on behavior, non-specific reward; Discrepancy between current behavior and goal; action coping	Education, training, incentivization, persuasion, empowerment // Psychological capabilities, reflective and automatic motivation
Anti-sugar mountain quiz	Gamification	Information about health consequences; Instruction on how to perform a behavior	Education // Psychological capabilities, reflective motivation
Informative Feedback	Tips are motivational (low-cost and easy tips); inspiration through recipes, food alternatives; positive, encouraging feedback	Feedback on behavior, non-specific reward, instructions on how to perform a behavior, action planning, action coping	Education, persuasion, incentivization, training, empowerment // Reflective and automatic motivation; psychological capabilities, physical and social opportunity

C2go app features	Target group preferences	BCTs	Intervention functions // COM-B components
Evaluative feedback	Positive, encouraging feedback	Verbal persuasion about capability, feedback on behavior	Persuasion, incentivization // Reflective and automatic motivation
Motivating feedback	Positive, encouraging feedback	Verbal persuasion about capability, feedback on behavior	Persuasion, incentivization // Reflective and automatic motivation
Quiz	Features for use when bored/individual time of usage; gamification	Information about health consequences; Instruction on how to perform a behavior	Training // Psychological capabilities
Points and level	Maintaining suspense, incentives, gamification	Non-specific reward	Incentivization // Reflective and automatic motivation
"Infothek" (with different topics)	Maintaining suspense; Customization/individual needs; nutrition education/information (e.g. more food information is asked for); interesting content, incentives, nutrition information, interests in topics as food waste, beauty, sports nutrition, recipes, food information, health	Non-specific reward	Incentivization // Reflective and automatic motivation
Community and leaderboard	Used by peers; social comparison	Social comparison	Persuasion // Automatic and reflective motivation
Avatar	Customization/individual needs; social acceptance/used by peers	Social support	Empowerment // Social opportunity
Reminders	Support through reminder; customization/individual needs	Prompts/cues	Education // Psychological capabilities, reflective motivation
Considered	Android operating system, everyday relief through mobile phone/app usage; clear layout; low food expenses; carefree eating according to taste and preferences, without waiver; satisfy physiological needs; high range of functions; no advertisement; no costs; smooth running		
Web app	Flat rates; low memory usage		
Imprint	Data protection and privacy		
[a] includes in Prototype II: introductory question, profile settings, tutorial; Prototype I: profile settings and tutorial only			
